# About Face: Seeing the Talker Improves Spoken Word Recognition but Increases Listening Effort

**DOI:** 10.5334/joc.89

**Published:** 2019-11-22

**Authors:** Violet A. Brown, Julia F. Strand

**Affiliations:** 1 Washington University in St. Louis, Department of Psychological and Brain Sciences, US; 2 Carleton College, Department of Psychology, US

**Keywords:** Auditory word processing, Multi-sensory perception, Attention

## Abstract

It is widely accepted that seeing a talker improves a listener’s ability to understand what a talker is saying in background noise (e.g., Erber, 1969; Sumby & Pollack, 1954). The literature is mixed, however, regarding the influence of the visual modality on the listening effort required to recognize speech (e.g., Fraser, Gagné, Alepins, & Dubois, 2010; Sommers & Phelps, 2016). Here, we present data showing that even when the visual modality robustly benefits recognition, processing audiovisual speech can still result in greater cognitive load than processing speech in the auditory modality alone. We show using a dual-task paradigm that the costs associated with audiovisual speech processing are more pronounced in easy listening conditions, in which speech can be recognized at high rates in the auditory modality alone—indeed, effort did not differ between audiovisual and audio-only conditions when the background noise was presented at a more difficult level. Further, we show that though these effects replicate with different stimuli and participants, they do not emerge when effort is assessed with a recall paradigm rather than a dual-task paradigm. Together, these results suggest that the widely cited audiovisual recognition benefit may come at a cost under more favorable listening conditions, and add to the growing body of research suggesting that various measures of effort may not be tapping into the same underlying construct (Strand et al., 2018).

As anyone who has been to a noisy party can attest, seeing a talker’s face typically facilitates speech recognition. This “visual enhancement” has been robustly demonstrated across a wide range of listening conditions (e.g., Erber, 1969; Grant, Walden, & Seitz, 1998; Helfer & Freyman, 2005; Sumby & Pollack, 1954; Van Engen, Phelps, Smiljanic, & Chandrasekaran, 2014). Visual enhancement quantifies the extent to which visual information facilitates speech intelligibility, but the accuracy with which speech is recognized provides an incomplete picture of the difficulty of the task. That is, measures of word recognition accuracy do not capture information about the cognitive load associated with processing speech; listeners may be able to maintain high levels of word recognition accuracy when conversing in either a quiet room or at a cocktail party with competing speech and loud background noise, but the cognitive and attentional demands of listening in these two settings are quite different (see Mattys, Davis, Bradlow, & Scott, 2012 for a review).

These demands increase *listening effort*—“the deliberate allocation of mental resources to overcome obstacles in goal pursuit when carrying out a [listening] task” (Pichora-Fuller et al., 2016). Listening effort depends in part on transmission factors such as background noise, but also on listener motivation and fatigue (e.g., McGarrigle, Dawes, Stewart, Kuchinsky, & Munro, 2016), listener-intrinsic factors such as hearing impairment or cognitive decline, and source-related factors such as accented speech (Pichora-Fuller et al., 2016) or the presence of visual speech information. According to the Ease of Language Understanding (ELU) model, difficult listening conditions elicit a mismatch between the unfolding speech and representations of the corresponding words stored in the listener’s mental lexicon (Rönnberg et al., 2013). When these mismatches occur, cognitive resources must be recruited to resolve them. Given the common assumption that humans possess a limited pool of cognitive resources (Kahneman, 1973; Pashler, 1994), as more resources are recruited during more difficult listening tasks, fewer resources remain to quickly and accurately complete concurrent tasks or encode information into memory.

Given the multi-modal nature of speech and potentially negative consequences of effort (McGarrigle et al., 2014), the relationship between listening effort and audiovisual speech processing has received increasing attention in recent years (Fraser et al., 2010; Gosselin & Gagné, 2011a; Mishra, Lunner, Stenfelt, Rönnberg, & Rudner, 2013a, 2013b; Sommers & Phelps, 2016). Existing models such as the ELU model and the Framework for Understanding Effortful Listening (FUEL) do not explicitly address how adding a visual signal affects listening effort, and arguments could be made for several patterns of data. Seeing a talking face in addition to hearing the voice might be expected to *increase* effort (e.g., Fraser et al., 2010) if simultaneously monitoring two modalities and combining them into a unified percept demands greater cognitive load than monitoring a single channel. Conversely, being able to see as well as hear a talker may instead be expected to *reduce* effort (e.g., Sommers & Phelps, 2016); the complementary information provided by the visual signal may limit the lexical search space and facilitate mapping the input onto phonetic representations in memory, thereby lessening the cognitively demanding process of lexical competition (Kuchinsky et al., 2013; Wagner, Toffanin, & Başkent, 2016).

It is currently difficult to adjudicate between these possibilities, as the literature appears to contain support for every claim possible about the difference in effort required for audiovisual (AV) versus audio-only (A-only) speech processing: greater effort for AV than A-only speech (Fraser et al., 2010; Gosselin & Gagné, 2011a), less effort for AV than A-only speech (Sommers & Phelps, 2016), equivalent effort for AV and A-only speech (Keidser, Best, Freeston, & Boyce, 2015), and differential effects of effort based on the conditions of testing (Mishra et al., 2013a). A survey of the existing research suggests at least two possible explanations for the inconsistencies across studies.

First, the relationship between listening effort and adding visual information may interact with task difficulty such that any reductions in listening effort associated with seeing the talker are only observed in difficult listening conditions (see Mishra et al., 2013a). That is, simultaneously processing auditory and visual information may increase listening effort, either because a moving face is distracting and diverts attention from processing the auditory speech, or because trying to integrate information from two sources requires more cognitive resources (Alsius, Navarra, Campbell, & Soto-Faraco, 2005). However, this cost may have the potential to be offset by the beneficial complementary information provided by the visual signal (Grant & Walden, 1996). This interpretation implies that in easy listening conditions (in which the auditory input is fully intelligible), visual information should increase effort because these conditions incur two-channel processing costs (via distraction, costs associated with integrating, or some other mechanism) with only a minimal benefit of reduced lexical competition. In adverse conditions, however, the visual input reduces competition from simultaneously activated lexical candidates (Tye-Murray, Sommers, & Spehar, 2007), which may therefore reduce overall effort. The reduction in effort specifically in difficult listening conditions is consistent with the general claims of the ELU, as the benefits afforded by the visual signal would reduce the mismatch between the acoustic input and stored mental representations of words, so fewer cognitive resources would need to be recruited to resolve the mismatch.

Indeed, in relatively easy listening conditions in which visual information is not likely to narrow the response set (e.g., closed-set tasks with phonologically dissimilar response options), visual information tends to increase effort (Fraser et al., 2010; Gosselin & Gagné, 2011a), whereas in difficult listening conditions, visual input may decrease effort (see Mishra 2013a). Studies that show no difference in the effort required to process A-only versus AV speech (e.g., Keidser et al., 2015; Picou & Ricketts, 2014) may have found conditions in which the costs and benefits of visual information are approximately equivalent. Further, prior studies have used speech materials with differing levels of complexity—including both words (Sommers & Phelps, 2016) and sentences (Fraser et al., 2010; Gosselin & Gagné, 2011a)—and it may be that any processing load associated with adding the visual signal accumulates with increased complexity. Studies also differ in how masking noise is applied to the A-only and AV conditions; some studies fix the level of the background noise in A-only and AV conditions, whereas others adjust the level of noise presented in the two conditions to result in equivalent levels of speech intelligibility in A-only and AV conditions (see Fraser et al., 2010 for an example of both techniques). Thus, the inconsistent findings in prior studies may be a function of the level of background noise (e.g., an easy versus a hard signal-to-noise ratio (SNR); whether SNR is fixed or manipulated to achieve a desired level of recognition accuracy), materials used (e.g., words versus sentences or open-set versus closed-set speech tasks), talker intelligibility, or some other factors that may affect task difficulty.

Alternatively or in addition, the contradictory findings may be due to the fact that effort has been operationalized differently across studies. Over two dozen tasks have been used to quantify listening effort (see Strand, Brown, Merchant, Brown, & Smith, 2018), but evidence is accumulating that these measures of listening effort are in fact tapping into different underlying constructs (Alhanbali, Dawes, Millman, & Munro, 2019; Strand et al., 2018). Indeed, measures of listening effort differ in their sensitivity to the addition of noise, their relationships with other measures of effort, and the extent to which they depend on underlying cognitive abilities (Strand et al., 2018). Therefore, the observed inconsistencies across studies may be a function of the different paradigms used to measure listening effort.

The most commonly used classes of behavioral measures of listening effort are dual-task and recall-based measures (Strand et al., 2018). In dual-task paradigms (see Gagné, Besser, & Lemke, 2017 for a review), participants are typically asked to listen to speech (the primary task) while completing a secondary task (e.g., responding to an unrelated vibrotactile stimulus or making a judgment about the speech). Slower response times to the secondary task are taken as an indication of greater effort. In recall-based tasks, participants are asked to listen to and encode speech into memory and later recall what they perceived. The rationale for using recall-based tasks to measure listening effort is that more difficult listening conditions require greater cognitive recruitment and therefore leave fewer resources available to encode speech into memory (Rabbitt, 1968). Although both dual-task and recall-based tasks are assumed to measure listening effort, they are weakly correlated with one another (Strand et al., 2018), which may suggest that they are sensitive to different features of listening effort. That is, dual-task paradigms quantify listening effort on-line as the speech unfolds, whereas recall paradigms assess the downstream effects of listening effort on memory of what was heard. Thus, the conflicting findings in the literature may be attributable to the fact that the different paradigms are measuring different aspects of listening effort.

In support of this claim, dual-task and recall measures have tended to render different patterns of results in studies assessing how seeing the talker affects listening effort. Critically, each of the studies that have shown reduced effort in AV conditions employed a recall rather than a dual-task paradigm to assess effort (e.g., Frtusova, Winneke, & Phillips, 2013; Mishra et al., 2013a, 2013b; Rudner, Mishra, Stenfelt, Lunner, & Rönnberg, 2016; Sommers & Phelps, 2016), whereas the results of dual-task studies have been more mixed (Fraser et al., 2010; Gosselin & Gagné, 2011a; Picou & Ricketts, 2014). Why might recall-based measures be more likely than dual-task measures to show reductions in listening effort for AV relative to A-only speech? One explanation is that visual input reduces listening effort, but these effects take time to unfold and therefore are only detectable at the longer time scales required of recall tasks. Conversely, it may be that the AV benefits for recall measures are instead driven by a more general recall benefit for stimuli presented in two modalities (where the visual signal is text or a picture rather than visual speech) compared to unimodal presentation (i.e., dual code theory; Danan, 1992; Mastroberardino, Santangelo, Botta, Marucci, & Olivetti Belardinelli, 2008; Thompson & Paivio, 1994). That is, although improved recall in AV compared to A-only conditions may be attributed to reductions in effort, it may instead be the result of richer representations when stimuli are presented in multiple modalities because bimodal stimuli are effectively encoded twice, and therefore have nothing to do with effort *per se*.

To summarize, the literature is mixed on how seeing in addition to hearing the talker affects listening effort, and variability in task difficulty and measures of listening effort make it difficult to generalize across studies. The current study aims to reconcile the discrepant findings in the literature by varying both the paradigm used to measure effort (dual-task and recall) and the listening difficulty (easy and hard SNRs). The dual-task and recall paradigms we employed used the same speech materials to ensure that any differences observed between experiments cannot be attributed to the specific words used or the linguistic unit of analysis (e.g., phonemes, words, or sentences).

The current study also used the same levels of background noise in the A-only and AV conditions, meaning that intelligibility was expected to be higher in the AV condition than the A-only condition. As described above, some prior work (e.g., Gosselin & Gagné, 2011a) has instead matched speech *intelligibility* in the AV and A-only conditions by increasing the level of background noise in the AV condition. In these circumstances, dual-task responses are typically slower in AV than A-only conditions (Gosselin & Gagné, 2011a), which has been interpreted as evidence that seeing a face increases effort. However, a challenge to this interpretation is that slower response times in the AV condition may instead be due to the higher levels of background noise. Indeed, adding background noise has been robustly shown to increase listening effort (Rabbitt, 1968; Strand et al., 2018). Given that increasing background noise in the AV condition makes it difficult to disentangle the effects of noise and visual input, the current study opted to set a consistent SNR for both conditions.

We hypothesized that when listening effort was assessed using a dual-task paradigm, the presence of a talking face would reduce listening effort to a greater extent in hard relative to easy listening conditions. Specifically, we hypothesized that in easy listening conditions, listening effort would be equivalent across A-only and AV conditions, but that in hard listening conditions, listening effort would be reduced for AV speech. This hypothesis is consistent with the ELU model’s claim that greater mismatch between input and representations (as is the case in the A-only relative to AV modality when the listening conditions are difficult) incurs greater cognitive load. In contrast, we hypothesized that when listening effort was operationalized using a recall paradigm, the visual modality would reduce listening effort in both easy and hard listening conditions because memory representations for bimodally encoded stimuli are perceptually richer than those for unimodally encoded stimuli.

## Experiment 1: Dual-Task (Audio-Only)

In line with prior work (Fraser et al., 2010; Gosselin & Gagné, 2011a), we used a vibrotactile dual-task paradigm to ensure that any observed effects could be attributed to effort rather than sensory interference. Given that we developed this task to measure listening effort, we began by assessing whether the vibrotactile task could detect differences in listening effort that result from changes in the level of the background noise in the absence of visual information.

### Method

All stimuli, raw data, and code for all experiments are available at https://www.osf.io/86zdp.

#### Participants

The pre-registration form for this experiment—which includes details about the power analysis we conducted to determine sample size, hypothesized results, and exclusion criteria—is available at http://www.osf.io/5yqe4/. We pre-registered a sample size of 26 individuals with normal or corrected-to-normal vision and normal hearing, but technical error resulted in data loss for two participants, so we ran a total of 28 participants aged 18–23 to arrive at a final sample size of 26. No participants met any of the pre-registered exclusion criteria. The study took approximately 30 minutes to complete and participants were compensated $5 for their time. For all studies reported here, participants gave written consent prior to beginning the experiment, and the Carleton College Institutional Review Board approved all research procedures.

#### Speech stimuli

200 consonant-vowel-consonant words were randomly selected from the English Lexicon Project database (Balota et al., 2007). Potentially offensive words and multiple homophonous entries were replaced. Stimuli were recorded by a female speaker with a non-distinctive regional accent at 16-bit, 44100 Hz using a Shure KSM-32 microphone with a plosive screen and were equalized on root-mean-square (RMS) amplitude using Adobe Audition. For each participant, 100 of these were randomly selected without replacement to appear in each of the two levels of listening difficulty. The level of the speech was held constant at approximately 65 dB SPL, and the level of the noise was set to 55 dB SPL in the easy condition (SNR = 10 dB), and 69 dB in the hard condition (SNR = –4 dB). These SNRs were selected to elicit a range of performance in both modalities while avoiding floor-level performance in the A-only modality in the hard SNR, and these particular levels were selected based on subjective assessments of difficulty as determined by the authors. The background noise consisted of speech-shaped noise generated in Praat (Winn, 2018) to match the long-term average spectrum of the target stimuli.

#### Vibrotactile stimuli

Vibrotactile stimuli consisted of a short (100 ms), medium (150 ms), or long (250 ms) pulse train presented to the index finger of each participant’s non-dominant hand. The custom-made apparatus delivering the pulse train consisted of a 3D-printed finger rest with a direct current vibration motor, controlled via the digital output of a Cedrus RB-740 buttonbox. During each trial, participants were presented a pulse train from the vibrotactile stimulator and were instructed to respond with their dominant hand by pressing one of three buttons on a buttonbox corresponding to what they perceived. The buttonbox was labeled such that the button corresponding to “short” always appeared on the left, “medium” in the middle, and “long” on the right. Presentation of the vibrotactile stimuli coincided with presentation of the speech stimuli, beginning between 60 ms before speech onset and 190 ms after speech onset, and varied randomly in intervals of 50 ms. This time window ensured that making the decision about the vibrotactile stimulus coincided with listening to the speech, and the variability was included so participants could not anticipate precisely when the vibrotactile stimulus would occur and thus had to continuously attend to the task. Given that the average word length was 523 ms, the vibrotactile pulses tended to end before the offset of the word, even for the longest pulses (250 ms) beginning as late as possible (190 ms after speech onset). The vibrotactile stimulator was placed on a sound-absorbing pad on the participant’s knee under the testing desk to dampen the sound of the vibrations. Given that participants wore headphones and there was background noise present during all conditions, the sound generated by the vibration was not audible.

#### Procedure

Participants completed three blocks of trials—one vibrotactile familiarization block, one block in the easy SNR (10 dB), and one block in the hard SNR (–4 dB). Participants always completed the familiarization block before the dual-task blocks, but the two dual-task blocks were presented in a counterbalanced order. During the familiarization block, participants were presented with two examples of each pulse length along with visual labels identifying them. The pulses were separated by 2,000 ms. Next, participants were presented 18 randomly intermixed pulses, six of each length, and were asked to classify each pulse as short, medium, or long as quickly and accurately as possible. If their accuracy at classifying the pulses during this familiarization block was worse than 75% (i.e., worse than 14/18 correct), the entire block, including the brief exposure phase, was repeated. This block was included to ensure that participants could accurately classify the pulses according to their duration before completing the vibrotactile and speech tasks concurrently.

In the remaining two blocks, participants were presented lists of 100 words, each of which occurred 2,000 ms following the response to the previous trial. Auditory stimuli were presented binaurally via Sennheiser HD 280 Pro headphones. Participants were instructed to repeat aloud each word they perceived, guessing when unsure, while simultaneously performing the vibrotactile task. They were informed that the word recognition task was most important, so they should focus their attention on that task, but were encouraged to complete the vibrotactile task to the best of their ability (Bourland-Hicks & Tharpe, 2002; Desjardins & Doherty, 2013; Downs, 1982; Strand et al., 2018). Participants were given a brief break between blocks. Prior to beginning the first experimental block, participants completed ten practice trials in the easy SNR. Noise was presented continuously throughout the task. Response times to the vibrotactile task were used as a measure of listening effort. We hypothesized that response times to the vibrotactile task would be slower in the hard compared to the easy SNR, indicating that the vibrotactile dual-task paradigm is sensitive to changes in effort.

### Results and Discussion

Data for all experiments were analyzed using linear mixed effects models via the *lme4* package (version 1.1.21) in R (version 3.5.2; Bates et al., 2014) and when appropriate, *p*-values from summary outputs for mixed effects models were obtained via the *lmerTest* package (version 3.1.0; Kuznetsova, Brockhoff, & Christensen, 2017). For this and all experiments, model comparisons were conducted using likelihood ratio tests, and nested models differed only in the fixed effect of interest. In each experiment, participants and items (words) were entered as random effects, and SNR was dummy coded (hard = 0, easy = 1). For all mixed effects models reported in this paper, we attempted to utilize the maximal random effects structure justified by the design (Barr, Levy, Scheepers, & Tily, 2013). In cases of non-convergence we utilized different control parameters and optimizers to enable convergence, and where appropriate, removed random effects based on contribution to the total variance. In cases of singularity and overfitting, we removed random effects that showed correlations with other random effects of 1.00 or –1.00, or those that contributed little to the total variance. Details regarding the random effects structure for each model can be found in the R scripts provided at the link above.

Before conducting the primary analyses, we removed individual trials with response times more than three median absolute deviations (MADs) below or above a participant’s median response time for a given condition (48 trials; 1.2% of the data). We opted to use MAD rather than standard deviation to determine which trials should be excluded because response time data tends to be right skewed, and means and standard deviations tend to be quite sensitive to outliers (see Leys, Ley, Klein, Bernard, & Licata, 2013 for a discussion of the benefits of using MAD as opposed to standard deviation to detect outliers). The final analysis consisted of 3,967 trials. We had also pre-registered that we would exclude trials with response times above 2,000 ms, but opted to remove that exclusion criterion before analyzing any data because the pre-registered MAD criterion should exclude trials with extreme response times, and a strict cutoff that is applied to all conditions might selectively remove a greater number of trials from conditions with slower response times. We removed the 2,000 ms exclusion criterion in Experiment 2 as well. In this experiment and in Experiment 2, we only analyzed response times to vibrotactile pulses that were correctly classified as short, medium, or long.

**Figure 1:**
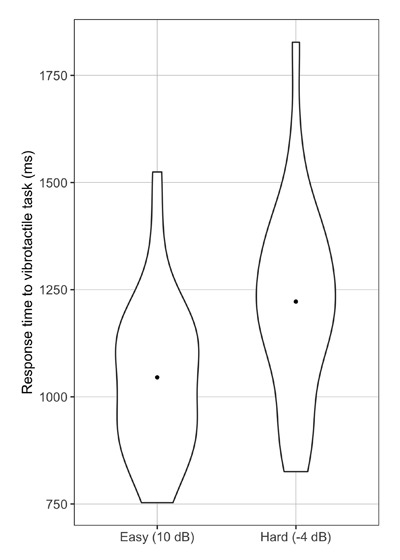
By-participant response times to the secondary vibrotactile task for the easy (left) and hard (right) conditions. The dot represents the mean response time in each condition, and the shape of each plot represents the distribution of responses across participants.

Participants correctly identified an average of 91% of words in the easy SNR and 47% in the hard SNR, indicating that the SNR manipulation resulted in a wide range of speech intelligibility (see Table 1). To determine whether response times to the vibrotactile task were slower in the harder SNR, we compared two nested models that differed only in the presence of the fixed effect for SNR. Note that we did not include vibrotactile pulse length as a random effect because this factor had only three levels. A likelihood ratio test indicated that the model including SNR as a fixed effect provided a better fit for the data than a reduced model without the SNR effect (*χ*^2^_1_ = 30.13; *p* < .001). Examination of the summary output for the full model indicated that on average, response times were an estimated 177 ms faster in the easy SNR than the hard SNR (β = –177.40, *SE* = 23.96, *t* = –7.40, *p* < .001; see Figure 1 and Table 1). These results indicate that this vibrotactile dual-task paradigm is sensitive to changes in noise, akin to other tasks assumed to measure listening effort (Strand et al., 2018).

**Table 1:** By-participant mean response times to correct responses on the vibrotactile task and mean accuracies at that task, as well as mean word recognition accuracies in the easy and hard SNRs and A-only and AV modalities for Experiment 1. Standard deviations are indicated in parentheses.

SNR	Accuracy on vibrotactile task (%)	Response time to vibrotactile task (ms)	Word recognition accuracy (%)

Easy	79.88 (10.89)	1,045 (180)	91.04 (3.46)
Hard	74.54 (12.82)	1,222 (226)	46.69 (6.12)

## Experiment 2: Dual-Task

Experiment 2 used a vibrotactile dual-task paradigm to quantify effort in A-only and AV conditions in the same SNRs as in Experiment 1. We hypothesized that in the hard SNR, response times to the secondary task would be faster in the AV than the A-only condition. In contrast, we hypothesized that response times to A-only and AV speech would not differ in the easy SNR because we expected that combining the auditory and visual information occurs automatically, and the simplicity of the speech task obviates the role of the visual signal in reducing lexical competition. In other words, we expected to observe an interaction between modality and listening condition, and hypothesized that planned comparisons would reveal decreased effort for AV compared to A-only speech in the hard but not the easy SNR.

Participants in both experiments also completed a lipreading task, because it may be that the addition of the visual signal only reduces effort in individuals with superior lipreading ability (i.e., there may be an interaction between modality and lipreading ability). Further, better lipreaders may only derive this benefit in difficult listening conditions (Picou, Ricketts, & Hornsby, 2011), so we may observe a three-way interaction between modality, SNR, and lipreading ability.

We conducted alternate versions of Experiments 2 and 3 prior to conducting those reported here (see Supplementary Materials). Those experiments used longer words that rendered ceiling-level recognition performance, and used an easier recall task that also rendered ceiling-level recall performance, which may mask the effects of interest. After analyzing those data, we opted to re-run the studies using consonant-vowel-consonant words (akin to those used in Experiment 1) that are more confusable and therefore more difficult. We also made the recall task more difficult by asking participants to recall four rather than three items. In the interest of transparency, the methods and results from those studies are available at https://www.osf.io/86zdp. The conclusions we were able to draw from those data were limited by ceiling-level performance but the general pattern of results that emerged was consistent with those reported here.

### Method

The pre-registration form for Experiments 2 and 3 is available at https://www.osf.io/8rejp.

#### Participants

57 Carleton College undergraduates participated in this experiment. Individuals who participated in Experiment 1 were ineligible to participate in Experiment 2 to avoid familiarity with the dual-task paradigm. A total of four participants were excluded from analyses for meeting at least one of our pre-registered exclusion criteria—three for poor accuracy at the word recognition task and one for poor accuracy at identifying the length of vibrotactile pulses. The final analysis included data from 53 participants; this sample size was necessary to reach our pre-registered stop criterion of at least 5,000 observations per condition. For this experiment and Experiment 3, participants were paid $10 for 1 hour of participation.

#### Speech Stimuli

Speech stimuli in both Experiments 2 and 3 consisted of words presented in A-only and AV conditions, in both easy and hard SNRs. In the AV conditions, the talker’s head, neck, and shoulders were visible, and in the A-only conditions, the screen was black. 544 consonant-vowel-consonant words (136 per condition) were randomly selected from the English Lexicon Project database (Balota et al., 2007) for use in this experiment. 20 additional words were selected for use as practice items (10 in Experiment 2, 13 in Experiment 3, some overlapping). Potentially offensive words, multiple homophonous entries, proper nouns, and plural nouns were replaced. One item was repeated due to experimental error, resulting in 544 total words but only 543 unique words. As in Experiment 1, the SNR in the easy condition was 10 dB and in the hard condition was -4 dB. All stimuli were recorded by the same female native English speaker without a strong regional accent using a Panasonic AG-AC90 camera and a Sony UWP-D11 Lavalier microphone system.

#### Lipreading Stimuli

Lipreading stimuli consisted of five Build-A-Sentence (BAS) lists, each of which contains 12 sentences and 36 keywords (see Tye-Murray et al., 2008; Tye-Murray, Spehar, Myerson, Hale, & Sommers, 2016). An additional six sentences were chosen from another BAS list for use as practice items. In the BAS task, participants are presented with sentences following the same general structure, and must select target words from a closed set of 36 nouns. All sentences follow the structure: “The *boy* and the *dog* watched the *cook* and the *moose*,” where each sentence contains between two and four nouns—at least one before the verb “watched” and one after—that appear only in the italicized positions. Each list contains three of each of the four sentence frames, and each target word appears in each list exactly once; thus, participants were presented a total of 180 target words (36 words * 5 lists). However, due to experimental error, one of the target words did not appear in the word bank, so that word was removed from analysis in each of the five lists for all participants. Thus, the total number of words participants could receive credit for lipreading was 175. The lipreading stimuli were recorded by the same speaker who recorded the A-only and AV stimuli and were presented without background noise.

#### Vibrotactile stimuli

The vibrotactile stimuli were identical to those described in Experiment 1, except in this experiment the vibrotactile stimuli began between 0 and 250 ms before speech onset (varying randomly in intervals of 50 ms). This was an unintended deviation from Experiment 1 that was not identified until after study completion. However, given the length of the vibrotactile stimuli and the time required to process and respond to them via button press (average response times to the pulses were 1,058 ms, see “Results”), these processes still coincide with presentation of the speech.

#### Procedure

Participants completed six blocks of trials—one vibrotactile familiarization block, four dual-task blocks corresponding to each of the four conditions (A-only and AV in the easy and the hard SNR), and one BAS lipreading block. Participants always completed the familiarization block before the dual-task blocks, but the four dual-task blocks were presented in a counterbalanced order according to a Latin Square design. The 544 words were randomly divided into four lists of 136 words, and each list was used in each of the four conditions an equal number of times. The procedures for the vibrotactile task and familiarization block were identical to those described in Experiment 1. After completing the familiarization, participants completed ten practice trials in the AV condition at the easy SNR prior to beginning the task.

Participants completed the lipreading task last. In this task, they were presented with BAS stimuli followed by a screen containing the 36 possible words that could fill in the blank in a four-by-nine grid. After each sentence, participants were instructed to repeat aloud the sentence they think the talker said. Lipreading score was calculated as the proportion of words correctly perceived, irrespective of order. The order in which the lists were presented was pseudorandomized. Participants completed six practice trials prior to beginning.

### Results and Discussion

A total of 321 trials with extreme response times (as defined by the pre-registered MAD criterion) were removed (accounting for 1.46% of correct usable trials). Average accuracy at classifying vibrotactile pulses in each of the four conditions is reported in Table 2. The final analysis consisted of 21,679 response time trials; the condition with the fewest observations (A-only, hard) had 5,130 observations. The mean lipreading score was 38.47% (SD = 13.27%; range = 5.71%–66.86%).

**Table 2:** By-participant mean response times to correct responses on the vibrotactile task and mean accuracies at that task, as well as mean word recognition accuracies in the easy and hard SNRs and A-only and AV modalities for Experiment 2. Standard deviations are indicated in parentheses.

SNR	Accuracy on vibrotactile task (%)		Response time to vibrotactile task (ms)		Word recognition accuracy (%)	

	AV	A-only	AV	A-only	AV	A-only

Easy	79.80 (10.03)	77.90 (8.86)	1,047 (197)	998 (161)	97.21 (2.13)	92.22 (3.20)
Hard	75.35 (11.43)	72.34 (10.83)	1,111 (195)	1,089 (203)	75.15 (5.97)	39.96 (8.21)

We first built a full model predicting response times to correct responses on the vibrotactile task with SNR (dummy coded such that hard = 0 and easy = 1) and modality (dummy coded such that A-only = 0 and AV = 1) as fixed effects, including lipreading ability as a covariate. We then built two reduced models: one with SNR and lipreading ability (but not modality) as fixed effects, and the other with modality and lipreading ability (but not SNR) as fixed effects. A model that included SNR, modality, and lipreading ability provided a better fit for the data than a model without SNR (*χ*^2^_1_ = 23.39; *p* < .001), and average response times were an estimated 77 ms faster in the easy than the hard SNR (in the A-only condition; Table 3). The full model also provided a better fit than one without modality (*χ*^2^_1_ = 6.59; *p* < .01), with average response times in the AV condition being an estimated 34 ms slower than those in the A-only condition (in the hard SNR; Table 3).

**Table 3:** Coefficients, standard errors, t- or z-values, and p-values for the accepted models. t-values are reported for the continuously valued response times and z-values are presented for the binomially distributed word recognition accuracy data. See available code for details of the random effects structure for each model.

Model	Fixed effect of interest	*β*	*SE*	*t* or *z*	*p*

Response time ~ SNR + modality + lipreading	SNR	–76.91	14.32	–5.37	<.001
	modality	33.64	12.82	2.62	.01
Response time ~ SNR + modality + lipreading + SNR * modality	SNR * modality	28.76	6.71	4.29	<.001
	*Planned comparisons, Easy SNR*				
	modality	48.09	19.43	2.48	.02
	*Planned comparisons, Hard SNR*				
	modality	21.25	19.34	1.10	.28
Response time ~ SNR + modality + lipreading + SNR * modality * lipreading	SNR * modality * lipreading	295.70	50.81	5.82	<.001
	*Planned comparisons, Easy SNR*				
	modality * lipreading	55.23	149.02	0.37	.71
	*Planned comparisons, Hard SNR*				
	modality * lipreading	–265.27	143.80	–1.85	.07
Word recognition accuracy ~ SNR + modality + SNR * modality	SNR	3.88	0.09	41.16	<.001
	modality	1.94	0.08	24.18	<.001
	SNR * modality	–1.06	0.11	–9.59	<.001
Vibrotactile accuracy ~ SNR + modality	SNR	0.29	0.05	5.49	<.001
	modality	0.17	0.05	3.47	<.001

**Figure 2:**
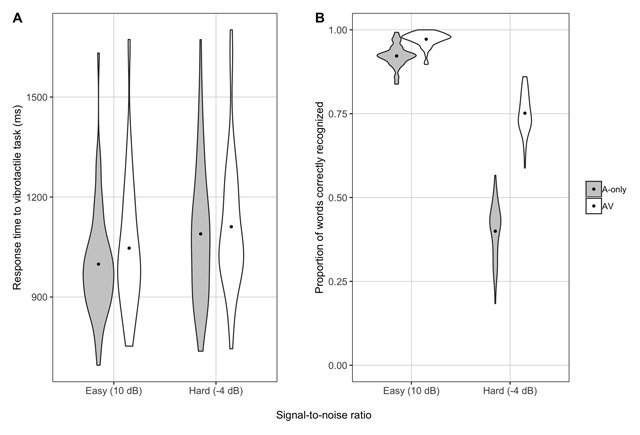
By-participant response times to the secondary vibrotactile task (A) and word recognition accuracy (B) in the easy and hard SNRs in the A-only and AV modalities in Experiment 2. The dot represents the mean response time (A) or accuracy (B) in each condition, and the shape of each plot represents the distribution of responses across participants.

We hypothesized that the effect of modality would differ as a function of listening difficulty such that seeing the talking face would provide more benefit in the hard SNR than the easy one. To test this, we built a model that included the interaction between SNR and modality, and compared it to a model without the interaction. The full model provided a better fit than the reduced model (*χ*^2^_1_ = 18.37; *p* < .001), and although response times tended to be faster in the A-only modality than the AV modality, the effect was larger in the easy condition (Table 3). Put another way, seeing the talker’s face increased effort overall, but the effect was more pronounced in the easy SNR (see Figure 2 and Table 2).

To further explore the nature of the SNR-by-modality interaction, we analyzed the data from the easy and hard SNRs separately and compared full models that included modality and lipreading ability as fixed effects to reduced models lacking the fixed effect for modality. In the easy SNR, the effect of modality was significant (*χ*^2^_1_ = 5.91; *p* = .02), with average response times an estimated 48 ms slower in the AV relative to the A-only condition (Table 3). The effect of modality was not significant in the hard SNR (*χ*^2^_1_ = 1.22; *p* = .27).

Given our finding that adding the visual modality increases effort, we performed an exploratory analysis to ensure that listeners recognized more words in the AV compared to the A-only condition, and in the easy compared to the hard SNR (Erber, 1969; Sumby & Pollack, 1954; i.e., a manipulation check). That is, we wanted to ensure that the somewhat counterintuitive finding that adding the visual signal actually increases effort cannot be attributable to unsuccessful AV stimuli whereby participants actually have poorer recognition accuracy in the AV condition. This analysis consisted of 28,807 trials. We used generalized linear mixed effects models with a logit link function for this analysis because accuracy was binomially distributed (0 = incorrect; 1 = correct). In line with prior work, the main effects of SNR (*χ*^2^_1_ = 223.35; *p* < .001) and modality (*χ*^2^_1_ = 150.57; *p* < .001) were both significant, as was the interaction between them (*χ*^2^_1_ = 64.10; *p* < .001). Participants correctly identified more words in the AV than the A-only condition and in the easy than the hard SNR. The significant interaction between SNR and modality indicated that although word recognition accuracy was better in the AV modality, this effect was less pronounced in the easy SNR (see Figure 2). In other words, consistent with previous work, participants received more visual enhancement in the hard compared to the easy SNR, presumably because there is little room for improvement in the easy SNR due to a ceiling effect.

Visual inspection of Table 2 suggests that although processing AV speech leads to slower response times to the secondary task, it actually leads to numerically higher accuracy at classifying pulses as short, medium, or long. To determine whether accuracy at this task differs as a function of modality and SNR, we conducted a set of exploratory analyses with accuracy at the vibrotactile task as the dependent variable (0 = incorrect; 1 = correct). As in the word recognition analysis described above, we used generalized linear mixed effects models with a logit link function to analyze these data. This analysis consisted of 28,826 trials. The main effects of SNR (*χ*^2^_1_ = 23.81; *p* < .001) and modality (*χ*^2^_1_ = 10.97; *p* < .001) were both significant such that accuracy was higher in the easy SNR than the hard SNR and higher in the AV condition than the A-only condition (see Table 3).

The next set of pre-registered analyses sought to examine the extent to which lipreading ability moderates the relationship between modality and effort expenditure. If better lipreaders can extract speech information from the visual modality more easily, then perhaps the detrimental effects of adding the visual signal would not be apparent in good lipreaders. Contrary to this prediction, a model predicting response time with fixed effects for SNR, modality, lipreading ability, and a lipreading ability-by-modality interaction did not provide a better fit than a model without the interaction term (*χ*^2^_1_ = 0.26; *p* = .61).

**Figure 3:**
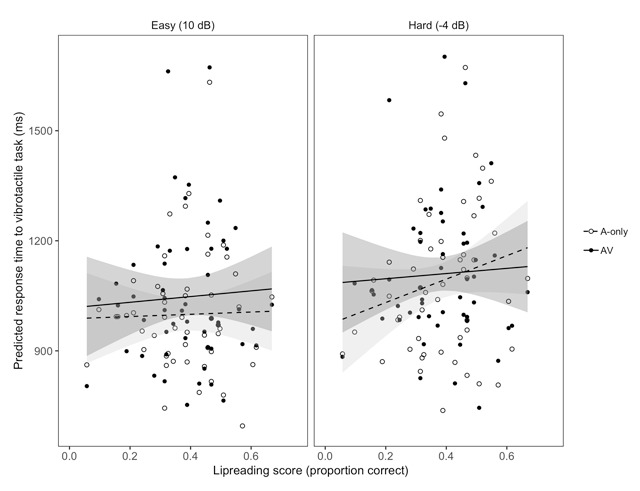
Model predictions and 95% confidence intervals for average response times to the vibrotactile task as a function of lipreading score, SNR, and modality. Each point represents the average response time for a given condition and participant.

We had also hypothesized that the effort-related benefit of superior lipreading ability may only manifest in difficult listening conditions, in which the visual modality has a greater capacity to benefit speech intelligibility, thereby reducing the effortful process of lexical competition. A model with the three-way interaction between SNR, modality, and lipreading ability provided a better fit for the data than a model with all two-way interactions but no three-way interaction (*χ*^2^_1_ = 33.84; *p* < .001). To examine the source of the interaction, we again analyzed each SNR separately, this time focusing on the interaction between modality and lipreading ability in each SNR. In the easy SNR, the model including the modality-by-lipreading interaction did not provide a better fit for the data (*χ*^2^_1_ = 0.14; *p* = .71). In the hard SNR, the model including the interaction also did not provide a better fit for the data (*χ*^2^_1_ = 3.42; *p* = .06). Examination of the summary outputs for both full models indicated that though non-significant, the estimated beta coefficient for the interaction term was positive in the easy SNR but negative in the hard SNR (see Table 3). This discrepancy explains the three-way interaction described above—although the estimate for the interaction parameter is not significantly different from zero in either SNR, these parameters differ significantly from one another (Figure 3). Unexpectedly, it appears that this finding regarding lipreading ability is driven by the A-only rather than the AV condition; we discuss this in more detail below.

Experiment 2 demonstrated that response times to the vibrotactile task were slower when speech was presented in higher levels of background noise or when speech was presented in the AV modality relative to the A-only modality. The finding that response times are slower when the SNR is poorer has been shown in a number of previous studies (Picou & Ricketts, 2014; Picou, Ricketts, & Hornsby, 2013; Sarampalis, Kalluri, Edwards, & Hafter, 2009; Strand et al., 2018), and together with Experiment 1 indicates that the vibrotactile task is sensitive to changes in noise. The finding that response times are slower in the AV relative to the A-only condition is consistent with prior research using vibrotactile dual-task paradigms to assess effort across modalities (Fraser et al., 2010; Gosselin & Gagné, 2011a), and suggests that AV speech processing generates greater cognitive load than A-only speech processing when noise level is matched.

Crucially, this experiment also demonstrated that the detrimental effect of the addition of the visual modality on effort was more pronounced in the easy SNR. Indeed, planned comparisons indicated that participants expended significantly more effort in the AV than A-only modality in the easy but not hard SNR. Though we had hypothesized that in the easy condition AV and A-only speech would require equivalent effort, and in the hard condition AV speech would require less effort than A-only speech, the observed results are consistent with the hypothesis that the visual signal has a greater capacity to reduce effort in more difficult listening conditions. That is, we had not expected that adding the visual signal would increase effort, but the interaction suggests that there may exist a pair of SNRs for which A-only and AV speech require equivalent effort in the easier condition, and AV speech actually requires less effort than A-only speech in the hard condition (i.e., the magnitude and direction of the modality effect depends upon the specific SNRs chosen).

We attribute this effect to the complementary nature of the visual speech signal (Grant & Walden, 1996). Although processing AV speech may initially incur greater cognitive load than processing A-only speech, in difficult listening conditions the visual modality also has the benefit of reducing the cognitively demanding process of lexical competition (Kuchinsky et al., 2013). In contrast, in easy listening conditions individuals benefit less from the visual signal than they do in difficult listening conditions (as demonstrated by the significant interaction between SNR and modality in our word recognition analysis), so incorporating the visual modality is costly without the benefit associated with a reduction in lexical competition. As a result, the increase in effort associated with the addition of a talking face is greater in easy than in hard listening conditions.

In this experiment, we used the same SNRs for A-only and AV speech to avoid introducing variation in SNR as a potential confound in the interpretation of the modality effect. That is, in the current study the only methodological difference between the A-only and AV conditions was the presence of a talking face. However, a by-product of this choice is that word recognition scores also differ between conditions—the same SNR results in poorer performance in the A-only than the AV condition. If we had instead opted to manipulate SNR such that A-only and AV word recognition accuracy were equated (e.g., by presenting the A-only hard condition at a more favorable SNR or the AV hard condition at a less favorable SNR), the two conditions would be matched on recognition accuracy, but would have different levels of background noise (see Gosselin & Gagné, 2011a for an example). Increasing the SNR in the A-only condition (or decreasing the SNR in the AV condition) to match performance in the two modalities would be expected to result in a larger difference in listening effort between A-only and AV speech particularly in the hard condition. As mentioned in the introduction, we opted to instead equate SNR to isolate the effect of modality on listening effort. That is, if we had equated performance across modalities, it would be difficult to discern whether any observed effects are attributable to the fact that we manipulated SNR or manipulated modality. However, future studies should address how fixing SNR versus recognition accuracy affects measures of LE and readers should be aware that this seemingly minor methodological choice has the potential to render large differences in study outcomes.

A somewhat puzzling finding from this experiment is that though response times to the secondary task were slower when the primary task involved recognizing words in the AV relative to the A-only modality, an exploratory analysis revealed that accuracy at identifying the vibrotactile pulses was actually *better* when the words were presented in the AV modality. One possible explanation for this finding is that the effort required to process AV speech slows response times to the secondary task, and this slowing allows participants to have more time to accurately perform that task. That is, there may be a speed-accuracy tradeoff during AV speech processing whereby slower responding improves accuracy. Further, it is unclear why this speed accuracy tradeoff exists for the modality comparison but not for the SNR comparison—participants were both slower and less accurate at the vibrotactile task in the hard relative to the easy SNR. This may be because the increases in effort that occur when listeners process AV relative to A-only speech are sufficiently small that they slow response times without affecting accuracy at the secondary task. However, increasing the level of the background noise may make the speech task difficult enough that participants can no longer perform both tasks accurately. In other words, when listening conditions are sufficiently difficult, the speech task may actually exhaust the listener’s pool of cognitive resources, so resources that otherwise would have been allocated to performing the secondary task accurately instead must be used to complete the speech task, resulting in poorer accuracy at both tasks. This explanation is speculative, however, so future research should explicitly address any speed-accuracy tradeoffs that may exist in research assessing listening effort for AV speech using a dual-task paradigm.

## Experiment 3: Delayed Recall

In Experiment 3, we used a recall paradigm to assess effort, with the expectation that the recall task would lead to a different pattern of results than the dual-task paradigm because bimodal encoding is likely to benefit recall in both easy and hard listening conditions (Thompson & Paivio, 1994). We hypothesized that participants would show superior recall for AV than A-only speech in both SNRs, and we expected that there would not be an interaction between modality and noise. In support of this prediction, Rudner and colleagues (2016) showed that young adults had better free recall of words presented in an AV condition compared to an A-only condition, and this effect did not interact with noise (i.e., recall was better in AV conditions in both quiet and noise). However, if an interaction between modality and noise emerged, we hypothesized that planned comparisons would reveal a significant positive effect of modality in both listening conditions, but a larger effect in the hard SNR because these are the conditions in which the visual signal can reduce lexical competition, and therefore listening effort, the most.

### Method

#### Participants

54 Carleton College undergraduates participated in this experiment. Two participants were excluded from subsequent analyses because they met at least one of our pre-registered exclusion criteria (one was eliminated for poor recall and one was eliminated for low response rates to words). We only analyzed data from the first 50 participants.

#### Speech stimuli

The speech stimuli in Experiment 3 were identical to those used in Experiment 2.

#### Procedure

Participants completed five blocks of trials—four recall blocks corresponding to each of the four conditions (A-only and AV; easy and hard) followed by a BAS lipreading block. As in Experiment 2 the four experimental blocks were presented in a counterbalanced order according to a Latin Square design, and the lipreading block was always completed last. The lipreading task was identical to that in Experiment 2.

Recall was measured using a running memory task akin to that used by McCoy and colleagues (2005; see also Sommers & Phelps, 2016; Strand et al., 2018). Participants were presented with streams of spoken words separated by 1,000 ms and were asked to repeat aloud what they perceived after each word, guessing when unsure. After lists of varying lengths, they were instructed to repeat aloud the final four words they had perceived when prompted with four asterisks (to avoid exposure to additional verbal material; McCoy et al., 2005).^1^ The next trial was initiated with a key press or after eight seconds. Participants heard 16 pseudorandomized lists of words of lengths 5 through 12 (two of each length) in each of the four conditions. Each of the 544 words was randomly assigned to lists and hand-checked to avoid semantic relatedness, and each participant was presented with the same lists of words, presented in the same pseudorandomized order within each list. Lists were counterbalanced across conditions such that each list appeared in every condition. Participants were given brief breaks between blocks. Due to experimental error, one list was repeated for 15 participants. The second repetition of that list was excluded from analysis only for those participants.

Performance on the recall task was determined based on the number of words correctly recalled in the 3- and 4- back positions (McCoy et al., 2005; Sommers & Phelps, 2016; Strand et al., 2018), so there were 32 critical words per condition (2 words * 16 lists). Note that participants repeated four words aloud for each list, but only two of those words were included in the recall analysis. Given that the SNR in the hard listening condition was expected to adversely affect recognition accuracy, credit was given when words were recalled as they were perceived; that is, if a participant initially perceived the word incorrectly but then recalled that incorrect word, it was counted as correct (Johnson, Xu, Cox, & Pendergraft, 2015; Ng, Rudner, Lunner, Pedersen, & Rönnberg, 2013; Pichora-Fuller, Schneider, & Daneman, 1995; Sarampalis et al., 2009). If the participant did not repeat a word but later recalled it correctly, it was counted as correct. If they neither repeated nor recalled the word, it was counted as incorrect. Participants completed practice that consisted of one list of five words and one list of eight words, all in the AV condition in the easy SNR prior to beginning the first experimental block. If participants appeared not to understand the task (e.g., failing to repeat each word aloud), the practice block was repeated.

### Results and Discussion

The analyses below mirrored those described in Experiments 1 and 2, except here the dependent variable was recall of the 3- and 4-back words rather than response time. Because recall accuracy was binomially distributed (i.e., recall was scored as 1 for correct or 0 for incorrect), data in Experiment 3 were analyzed using generalized linear mixed effects models with a logit link function.

We built a full generalized linear mixed effects model containing SNR, modality, and lipreading ability as fixed effects, and compared it to each of two reduced models that lacked either SNR or modality as a fixed effect. The full model provided a better fit for the data than the model that lacked SNR (*χ*^2^_1_ = 37.97; *p* < .001); the odds of correctly recalling a word increased by a multiplicative factor of 1.62 in the easy compared to the hard SNR (in the A-only condition, *β* = 0.48, *SE* = 0.07, *z* = 7.07 , *p* < .001). This SNR effect on recall is consistent with the results of previous research (Johnson et al., 2015; Ng et al., 2013; Pichora-Fuller et al., 1995; Rabbitt, 1968; Sarampalis et al., 2009; Strand et al., 2018), and given that participants were granted credit for recalling words as they were perceived, this finding cannot be driven by poorer intelligibility in the hard SNR.

**Figure 4:**
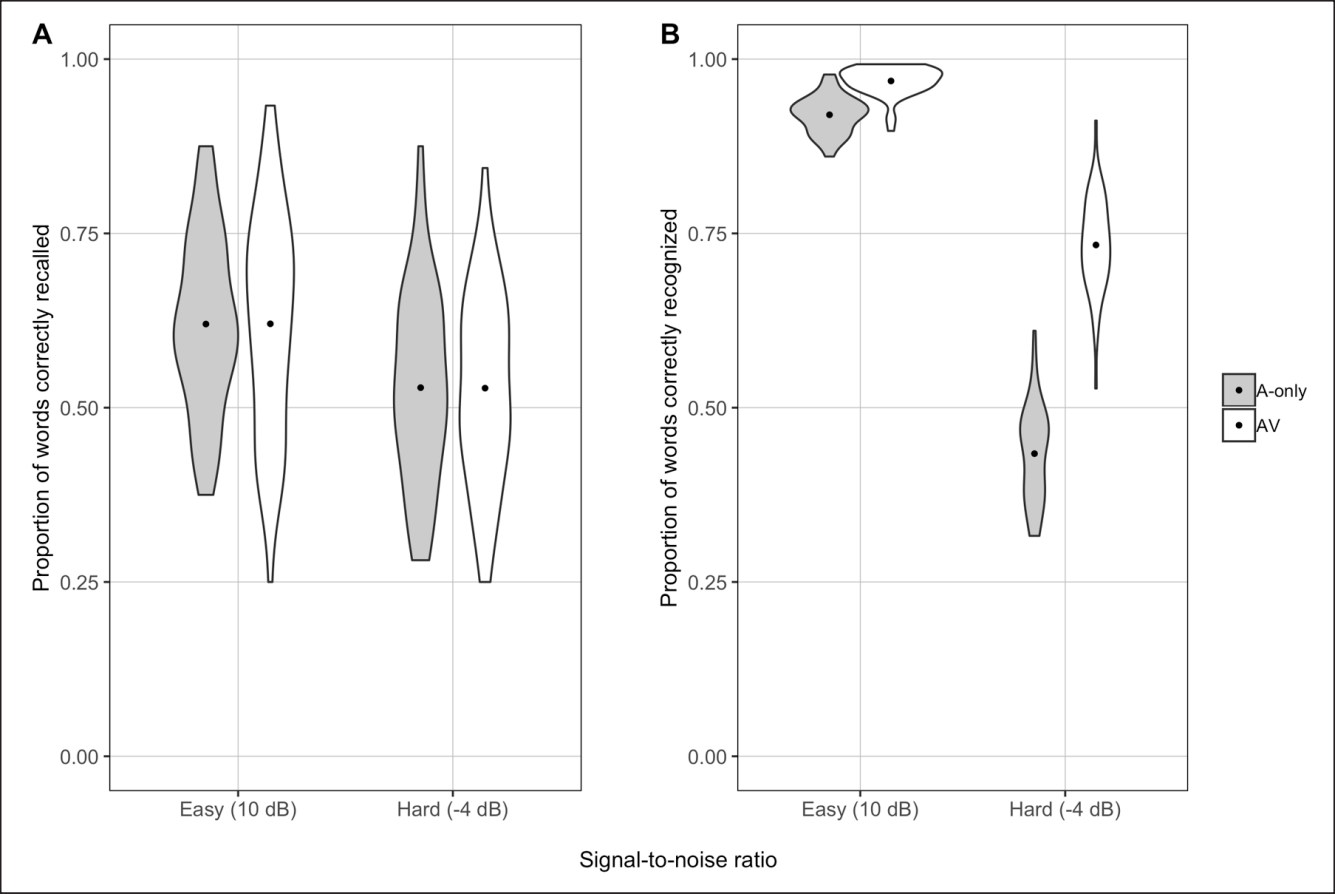
By-participant recall of the 3- and 4-back words (A) and overall word recognition accuracy (B) in the easy and hard conditions in the A-only and AV modalities in Experiment 3. The dot represents the mean recall (A) or accuracy (B) in each condition, and the shape of each plot represents the distribution of responses across participants.

Another likelihood ratio test indicated that the full model did not provide a better fit for the data than a reduced model without modality (*χ*^2^_1_ = 0.00; *p* = .99), suggesting that we found no evidence for an effect of modality on recall. A model containing the interaction between SNR and modality also did not provide a better fit than one without it (*χ*^2^_1_ = 0.01; *p* = .91). Thus, participants recalled fewer words in the hard SNR than the easy one, but there were no recall differences between A-only and AV speech, and we found no evidence for an SNR-by-modality interaction (see Figure 4 and Table 4). Given that we showed better speech intelligibility in easier SNRs and in the AV modality in Experiment 2, we opted not to perform this exploratory analysis again on the same words (but see Figure 4 for a plot of word recognition accuracy in Experiment 3).

**Table 4:** By-participant mean recall and speech recognition accuracy in the easy and hard SNRs as well as the A-only and AV modalities for Experiment 3. Standard deviations are indicated in parentheses.

SNR	Recall accuracy (%)		Word recognition accuracy (%)	

	AV	A-only	AV	A-only

Easy	62.03 (15.75)	62.00 (12.51)	96.87 (2.07)	92.01 (2.63)
Hard	52.80 (13.55)	52.88 (13.67)	73.34 (6.77)	43.41 (6.83)

*Note*: Recall accuracy refers to the 3- and 4-back words, but word recognition accuracy refers to the average accuracy for all words.

The mean lipreading score was 37.53% (SD = 14.13%; range = 10.86%–69.71%). A likelihood ratio test indicated that the interaction between lipreading ability and modality was not significant (*χ*^2^_1_ = 0.26; *p* = .61). Finally, a model that also included a three-way interaction between SNR, modality, and lipreading ability did not provide a better fit for the data than the one lacking the three-way interaction but including all two-way interactions (*χ*^2^_1_ = 2.98; *p* = .08). Taken together, these analyses indicate that the ability to extract information from the visual modality does not moderate the relationship between modality and listening effort as measured using this running memory task.

## General Discussion

The current studies validated the use of the vibrotactile dual-task paradigm by showing slowed response times in the hard relative to the easy SNR, and then tested the effects of SNR (easy versus hard) and modality (A-only versus AV) using two commonly used measures of effort (a dual-task and a recall paradigm). The dual-task experiment showed that hard listening conditions require greater effort and that relative to A-only speech, AV speech is recognized more accurately but also requires greater effort. Further, we showed that the AV *disadvantage* for effort is less pronounced when the listening conditions are more difficult, consistent with the general claims of the ELU (Rönnberg et al., 2013). In contrast, performance on the recall task was affected by SNR (with poorer recall in the hard condition than the easy one) but did not differ for A-only versus AV presentation.

The finding that response times to the vibrotactile task were significantly slower in the AV relative to the A-only modality *only* in easy listening conditions is consistent with the claim that processing AV speech initially increases cognitive demand, but the reduction in lexical competition provided by the visual signal in difficult listening conditions partially offsets this cost. It is possible, however, that in sufficiently difficult listening conditions (i.e., those in which the visual modality can maximally benefit recognition), the modality effect may actually reverse such that less effort is expended in AV relative to A-only conditions.

Using a dual-task paradigm, Fraser and colleagues (Fraser et al., 2010) demonstrated that for a set SNR, processing A-only and AV speech required comparable levels of effort. In contrast, we showed greater effort for AV than A-only speech. One possible explanation for these conflicting results is that these previous studies used closed-set tasks with phonologically dissimilar response alternatives (e.g., the French words *trouvent, cherchent*) whereas the current study used an open-set task. Dual-task paradigms also differ in their sensitivity to changes in effort (Picou & Ricketts, 2014; Strand et al., 2018), and the previously used paradigms may not have been sensitive enough or the studies may not have been sufficiently powered to detect the effects.

In contrast to the results from the dual-task experiments, we observed no effect of modality when we assessed effort using a recall paradigm: recall was poorer in louder background noise but was unaffected by modality. In the scoring system we used, words that were initially misidentified but later recalled (as misidentified) were scored as correct. Although participants misheard more words in the A-only condition than the AV condition, their recall was not affected by modality. This finding is not likely to be attributable to the fact that we equated SNR across the A-only and AV modalities, as Picou and colleagues (2011) also failed to find an effect of modality on recall when intelligibility was equated across the two modalities by varying SNR. The lack of a modality effect here and in Picou et al. (2011) is somewhat puzzling in light of the finding that other forms of multimodal encoding enhance recall (Danan, 1992; Mastroberardino et al., 2008; Thompson & Paivio, 1994) as well as comprehension (Arnold & Hill, 2001). One possible explanation for these null results is that processing AV speech comes at a cost, as demonstrated in Experiment 2, but improved recall in AV settings offsets these costs, resulting in a modality effect that is indistinguishable from zero. That is, the detrimental effects of the visual modality on effort may cancel out the beneficial effects of the visual modality on recall.

Finding no evidence for a modality effect in the recall paradigm is consistent with Picou and colleagues’ (2011) findings but differs from the findings of Sommers and Phelps (2016), who used very similar materials to those employed here and found that recall of 2- and 3-back items was significantly better for AV than A-only speech. One possible reason our results differ from Sommers and Phelps’ (2016) may lie in the difficulty of the tasks. Although Sommers and Phelps (2016) presented stimuli without masking noise, recall accuracy for the 2- and 3- back positions was much lower than what we found; visual inspection of the figures indicates that recall accuracy for A-only speech was approximately 40% in that study, compared to 62% in the easy SNR in Experiment 3. This discrepancy may stem from another methodological difference: in the current study (as well as in Picou et al., 2011), participants repeated each of the words aloud during the running memory task so recognition accuracy could be assessed in addition to recall accuracy (Pichora-Fuller et al., 1995; Sarampalis et al., 2009), whereas participants in Sommers and Phelps (2016) did not, as that task was conducted without background noise. It is conceivable that asking participants to repeat words aloud may have led to greater task engagement or created stronger memory traces, which may have masked any potential modality effects on recall. Thus, future research seeking to clarify this discrepancy should assess the circumstances under which recall tasks show benefits for AV speech.

Regardless of the mechanisms, the discrepancy in the results of the dual-task and recall experiments clearly suggests caution in using various measures of effort interchangeably when researching the cognitive costs associated with processing AV speech (see also McGarrigle et al., 2014). This research supplements the growing skepticism in the literature on listening effort in the A-only modality regarding whether various measures of listening effort are tapping into the same underlying construct (see Gosselin & Gagné, 2011b; Johnson et al., 2015; Seeman & Sims, 2015; Strand et al., 2018 for demonstrations of nonexistent or low correlations among various measures of listening effort). The lack of consistency in the results suggests that our initial concerns about the use of recall paradigms in AV settings may be warranted, and dual-task paradigms may be better equipped to detect differences in effort between A-only and AV speech without confounding effort with other factors. Further, as discussed above, dual-task paradigms are intended to measure listening effort in real time, whereas recall paradigms extend over longer time periods and assess the downstream consequences of listening effort. Thus, researchers should consider which type of paradigm is more appropriate to answer the research question of interest.

An attractive feature of dual-task paradigms is that they have clearly separable components for speech recognition and effort, meaning that a participant can fail to identify a given word but still have a score for listening effort on that trial. The fact that the ability to provide a response on the effort task is not dependent on correctly identifying the word means that researchers do not have to introduce additional degrees of freedom by deciding how to account for reliance of the effort measure on speech intelligibility. For running memory tasks, however, recall is dependent upon the intelligibility of the words; participants cannot recall a word they did not hear. For this reason, we opted to give credit for words correctly recalled as they were perceived (Johnson et al., 2015; Ng et al., 2013; Pichora-Fuller et al., 1995; Sarampalis et al., 2009). An alternate method to account for intelligibility is to adjust 2- and 3-back scores according to 1-back performance, which is assumed to depend only on intelligibility and not on recall (Strand et al., 2018). Studies conducted in quiet, however, tend to not account for intelligibility at all (e.g., Sommers & Phelps, 2016). Although each of these methods may be reasonable choices, this decision can be avoided altogether by using response time to an unrelated secondary task to operationalize effort.

Further, the separability of the speech and secondary tasks in the dual-task paradigm used here allows for additional validation of the secondary task in the absence of speech—using a dual-task paradigm in which the secondary task involved making judgments about visually-presented numbers, Brown and Strand (2018) showed that the secondary task was sensitive to changes in the level of the background noise only when speech was present. That is, it was not the case that performance on the cognitive task in isolation was impaired by noise, suggesting that the dual-task paradigm they used was indeed detecting changes in listening effort, and not simply changes in noise. Although we used a different dual-task paradigm in the present study to avoid sensory interference, the results of Brown and Strand (2018) suggest that dual-task paradigms can detect changes in the amount of effort expended during the speech task, and not just the amount of background noise. However, future research should determine whether performance on the vibrotactile secondary task we used here is adversely affected by noise in the absence of speech.

Experiment 2 indicated that lipreading ability moderates the interaction between listening difficulty and modality such that in hard listening conditions, the better a participant’s lipreading ability, the less affected they are by the detrimental effects of adding the visual modality (see also Picou et al., 2011). Though generally consistent with our hypotheses, it is intriguing that the three-way interaction appears to be driven by the A-only rather than the AV condition; that is, the slope relating lipreading ability and effort is more strongly positive in the A-only condition than the AV condition. This is surprising because one would expect that lipreading ability would be more strongly related to effort in AV conditions, and that the relationship would be negative. It is unclear why these results emerged, but future research could assess the extent to which lipreading ability moderates the relationship between AV speech and effort, perhaps using a more sensitive and ecologically valid measure of lipreading ability.

The generalizability of this research may depend on both the participant group studied and the nature of the materials. The experiments reported here involved normal-hearing young adults, who experience less listening effort in typical listening situations than older adults or hearing-impaired individuals (Desjardins & Doherty, 2013; McCoy et al., 2005). Given differences among these populations in lipreading ability (Shoop & Binnie, 1979; Sommers, Tye-Murray, & Spehar, 2005) and listening effort (Gosselin & Gagné, 2011a; McCoy et al., 2005), future work should address the extent to which the results extend to other populations. The pattern of results reported here may also be specific to the type of masking noise used. Indeed, different masker types may incur different processing costs (see Koelewijn, Zekveld, Festen, Rönnberg, & Kramer, 2012), and the extent to which the visual modality benefits recognition may differ across masker types (Helfer & Freyman, 2005). Thus, the information provided by a talking face may differentially affect cognitive load depending on the masker type.

Several mechanisms may account for the increase in effort from the presence of a talking face. One possibility is that combining speech information from the auditory and visual modalities into a unified percept is a cognitively demanding process. Alternatively, this process may occur automatically, but simultaneously monitoring two information-rich channels—which may involve detecting and resolving incongruity, assigning percepts to phoneme categories, or other mechanisms—is resource intensive. Finally, AV speech may simply be more engaging or distracting than A-only speech, resulting in larger performance deficits in the secondary task when the visual modality is present. Regardless of the mechanisms, the finding that recognizing AV relative to A-only speech is more resource-intensive despite substantial recognition benefits suggests that in everyday conversation, although looking at the talker’s face improves speech recognition, this benefit comes at a processing cost. This paper provides the first evidence that relative to A-only presentation of speech, AV speech increases word recognition accuracy while also incurring a processing cost when effort is assessed using a dual-task paradigm.

## Data Accessibility Statement

All data, code, and stimuli are available at https://www.osf.io/86zdp.

## Additional File

The additional file for this article can be found as follows:

10.5334/joc.89.s1 Supplementary materials. The Supplementary Materials for this article consist of alternate versions of Experiments 2 and 3 that were conducted prior to those reported here. These alternate versions rendered ceiling-level speech identification performance and the recall task was easier than that reported above, but we have included them in the interest of transparency.

## Footnotes

Note that both McCoy et al. (2005) and Sommers and Phelps (2016) asked participants to repeat the final three words they had heard, but a previous version of this experiment following this methodology (see Supplementary Materials) rendered ceiling-level performance so participants in Experiment 3 were instead asked to recall the final four words to increase task difficulty.
