# Supplementary material for: About Face: Seeing the Talker Improves Spoken Word Recognition but Increases Listening Effort
Source: J Cogn. 2019 Nov 22;2(1):44. doi: 10.5334/joc.89 (PMC6873894; doi:10.5334/joc.89)
Supplement: Supplementary materials. — The Supplementary Materials for this article consist of alternate versions of Experiments 2 and 3 that were conducted prior to those reported here. These alternate versions rendered ceiling-level speech identification performance and the recall task was easier than that reported above, but we have included them in the interest of transparency. [file joc-2-1-89-s1.pdf]

Supplementary materials

Experiments S2 and S3 described here were conducted after Experiment 1 and prior to the Experiments 2 and 3 reported in the manuscript. The methods of S2 and S3 are identical to Experiments 2 and 3, respectively, with two exceptions: S2/S3 used longer length words (see "Speech Stimuli" below) than Experiments 2/3 and the running memory task in S3 asked participants to recall the final three words rather than final four, as was done in Experiment 3. S2 and S3 resulted in very high levels of word recognition accuracy and this ceiling-level performance complicated interpretation of some of the effects of interest. Therefore, subsequent changes were made to Experiments 2 and 3 reported in the main paper. Unless otherwise noted, S2 and S3 follow the conventions of Experiments 2 and 3.

The main findings of S2 and S3 are very similar to Experiments 2 and 3. Deviations in the results of the studies are noted explicitly in each discussion section.

Experiment S2

Method

Participants. 53 individuals participated in Experiment S2, none of whom participated in Experiment 1. Two participants were eliminated from analyses because they met at least one of our pre-registered exclusion criteria (one was eliminated for poor accuracy on the word recognition task, and one was eliminated for the same reason, but also met the criterion for poor accuracy on the vibrotactile task). Since we pre-registered 50 participants in this experiment (provided that we had at least 5,000 observations per condition) and we had 51 usable data files after exclusion, the last participant we ran was also excluded from analyses, so here we report data from 50 participants. The pre-registration form for Experiments S2 and S3—which contains details about the power analysis we conducted to determine sample size, our initial hypotheses, our analysis plan, and exclusion criteria—is available at https://www.osf.io/86zdp.

Speech Stimuli. Words consisted of no more than 4 syllables, at least 3 phonemes, and had log-frequencies greater than 2.5 (Brysbaert & New, 2009). Proper nouns, plural nouns, and potentially offensive words were excluded, and all verbs were in the present tense. Although we had used consonant-vowel-consonant words in Experiment 1, we opted to use longer words here to make the stimuli more representative of speech more generally.

Results

Prior to conducting the effort analyses, we removed individual trials that met our pre-registered MAD exclusion criterion. This removed 262 trials with extreme response times. The final analysis consisted of 20,796 correct response time trials; the condition with the fewest observations (A-only, hard) had 5,099 observations.

The full model provided a better fit for the data than a model lacking SNR; that is, the main effect of SNR was significant ($\chi^2_1 = 7.20$; $p = 0.007$) and examination of the summary output indicated that response times were an estimated 33 ms faster in the easy relative to the hard SNR ($\beta = -33.18$, $SE = 12.02$, $t = -2.76$, $p = 0.008$). The main effect of modality was also significant ($\chi^2_1 = 11.39$; $\boldsymbol{p} < 0.001$), with response times in the A-only modality being an estimated 44 ms faster than those in the AV modality ($\beta = 44.18$, $SE = 12.48$, $t = 3.54$, $p < 0.001$). A model that included the interaction between SNR and modality did not provide a better fit for the data than a model lacking the interaction term ($\chi^2_1 = 0.98$; $p = 0.32$), indicating that we found no evidence that the effect of SNR differed by modality (Figure 1; Table 1).

Table 1.
Mean response times to correct responses on the vibrotactile task and speech recognition accuracy in the easy and hard SNRs as well as the A-only and AV modalities in Experiment S2.

	Response time to vibrotactile task (ms)		Word recognition accuracy (%)	
SNR	AV	A-only	AV	A-only
Easy	1,056	1,014	99.16	98.74
Hard	1,093	1,048	91.97	84.47

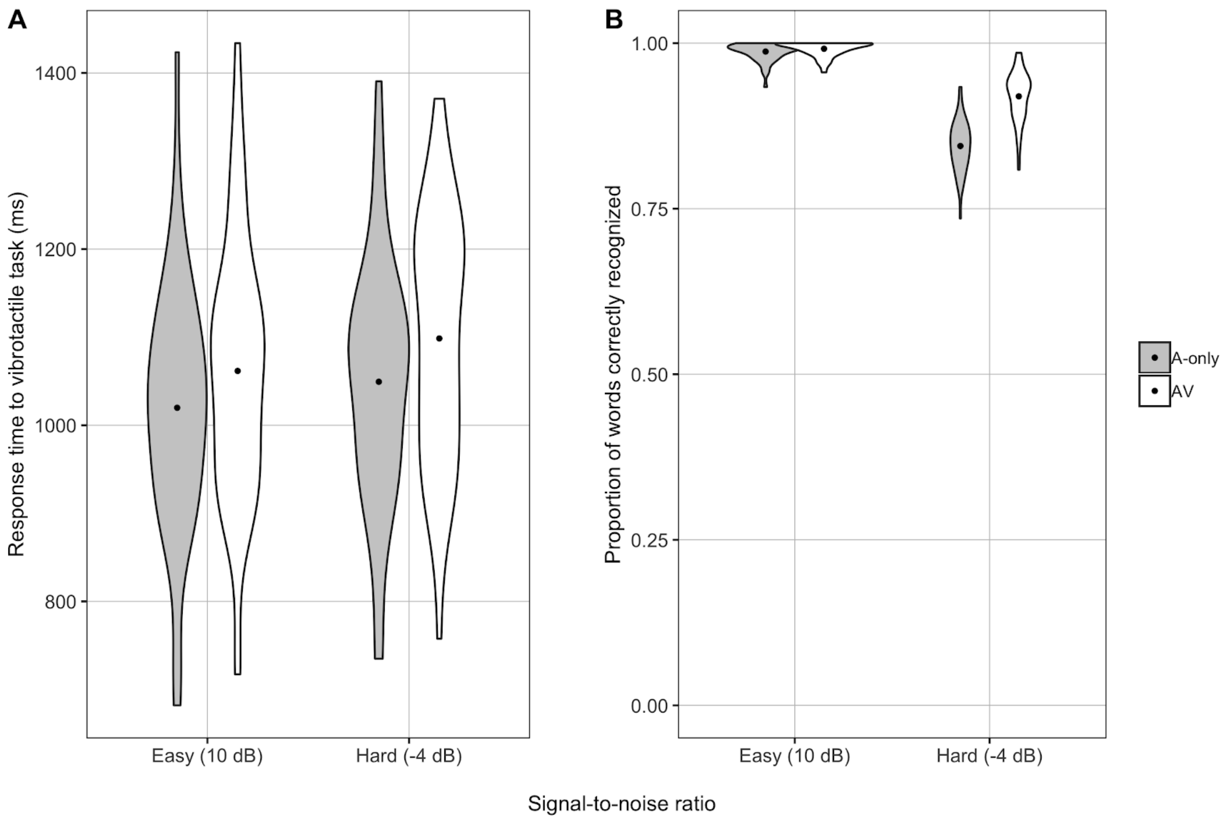

Figure 1. By-participant response times to the secondary vibrotactile task (A) and word recognition accuracy (B) in the easy and hard SNRs in the A-only and AV modalities in Experiment S2. The dot represents the mean response time (A) or accuracy (B) in each condition, and the shape of each plot represents the distribution of responses across participants.

We next performed an exploratory analysis of word recognition accuracy that consisted of 27,200 trials. The main effects of SNR (χ^2_1 = 133.44; $p < 0.001$) and modality (χ^2_1 = 36.54; $p < 0.001$) were both significant, as was the interaction between them (χ^2_1 = 6.90; $p = 0.009$). The odds of correctly recognizing a word increased by a multiplicative factor of 21.16 in the easy compared to the hard SNR (for the A-only condition; $\beta = 3.05$, $SE = 0.16$, $z = 19.53$, $p < 0.001$), and by a multiplicative factor of 2.25 in the AV compared to the A-only condition (in the hard SNR; $\beta = 0.81$, $SE = 0.10$, $z = 8.30$, $p < 0.001$). Finally, although word recognition accuracy was better overall in the AV condition, this effect was less pronounced in the easy SNR ($\beta = -0.65$, $SE = 0.22$, $z = -2.98$, $p = 0.003$). Thus, as would be expected, participants accurately identified more words in the easy than the hard SNR and in the AV than the A-only modality, and received more visual enhancement in the hard compared to the easy SNR.

To assess whether lipreading ability moderates the relationship between modality and effort expenditure, we built a model with SNR, modality, lipreading ability, and the lipreading ability-by-modality interaction as fixed effects. That model did not provide a better fit of the data than a model without the interaction (χ^2_1 = 0.07; $p = 0.79$).

Finally, we sought to assess whether the interaction between modality and listening difficulty is affected by lipreading skill (Picou & Ricketts, 2014). A model with the three-way interaction between SNR, modality, and lipreading ability did not provide a better fit for the data than a model without it (χ^2_1 = 0.34; $p = 0.56$).

Discussion

The results of Experiment S2 are quite similar to those of Experiment 2 reported in the main manuscript: response times to a vibrotactile classification task were slower when the primary speech recognition task was made more difficult by increasing the level of the background noise, and when the speech was presented in the AV relative to the A-only modality. The results of Experiments 2 and S2 were largely overlapping, but differed in two notable ways.

First, in Experiment 2, but not S2, we found a significant interaction between SNR and modality. This null effect in S2 is likely attributable to the simplicity of the task; that is, the speech task was easy enough in the A-only condition, even in the hard SNR, that the visual modality could not reduce lexical competition enough to reduce listening effort. Short words tend to have more neighbors than long words (Pisoni, Nusbaum, Luce, & Slowiaczek, 1985), and because Experiment 2 included only consonant-vowel-consonant words but Experiment S2 included longer words, the stimuli in Experiment 2 may have been more affected by noise. Indeed, the difference in word recognition accuracies between the easy and hard SNRs for A-only trials was much larger in Experiment 2 than in Experiment S2. It therefore appears that the difficulty of the listening task in Experiment 2 necessitated the recruitment of more cognitive resources than were required to recognize the longer words in Experiment S2, which may explain the discrepancy in the results across the two experiments.

A second difference is that in Experiment 2 (but not S2), a three-way interaction emerged between lipreading ability, SNR, and modality. We again attribute this to the difference in the difficulty of the speech tasks across the two experiments. In Experiment S2, the speech task was sufficiently easy that participants had near ceiling-level performance, even in the hard SNR in the A-only modality. These high levels of intelligibility across all conditions mean that the effects of SNR, modality, and lipreading ability have little opportunity to influence listening effort. In contrast, the speech recognition task in Experiment 2 was sufficiently difficult that it enabled these effects to emerge, so better lipreaders likely had greater opportunity to benefit from the addition of the visual signal than in Experiment S2.

Experiment S3: Recall Paradigm

Method

Participants. The task and stimulus materials were completely different in Experiment 1 and Experiment S3, so participants who completed the Experiment 1 were allowed to take part in Experiment S3. 52 individuals participated in this experiment, 12 of whom had participated in Experiment 1. Data

from two participants were excluded from analysis because they met at least one of our pre-registered exclusion criteria (one was eliminated for poor recall, and one was eliminated for poor word recognition accuracy). We therefore report data from 50 participants.

Stimuli. The speech stimuli were identical to those used in Experiment S2.

Procedure. The procedure was identical to Experiment 3 reported in the main manuscript, with the exception that participants were only asked to recall the final three (as opposed to four) words in each list, meaning that the analysis was conducted on 2- and 3-back words.

Results

The effect of SNR was significant ($\chi^2_1 = 5.07$; $p = 0.02$), and the odds of correctly recalling words increased by a multiplicative factor of 1.39 in the easy compared to the hard SNR (in the A-only condition; $\beta = 0.33$, $SE = 0.10$, $z = 3.34$, $p < .001$). The effect of modality was not significant ($\chi^2_1 = 0.08$; $\boldsymbol{p} = 0.78$). Finally, a model that included the interaction between SNR and modality did not provide a better fit than a model without the interaction ($\chi^2_1 = 2.08$; $\boldsymbol{p} = 0.15$), indicating no evidence that the effect of modality on recall differed as a function of listening difficulty (Figure 2; Table 2).

Table 2.
Recall and speech recognition accuracy in the easy and hard SNRs in the A-only and AV modalities.

	Recall accuracy (%)		Word recognition accuracy (%)	
	AV	A-only	AV	A-only
Easy	89.25	88.75	99.28	99.07
Hard	86.56	88.24	93.63	85.32

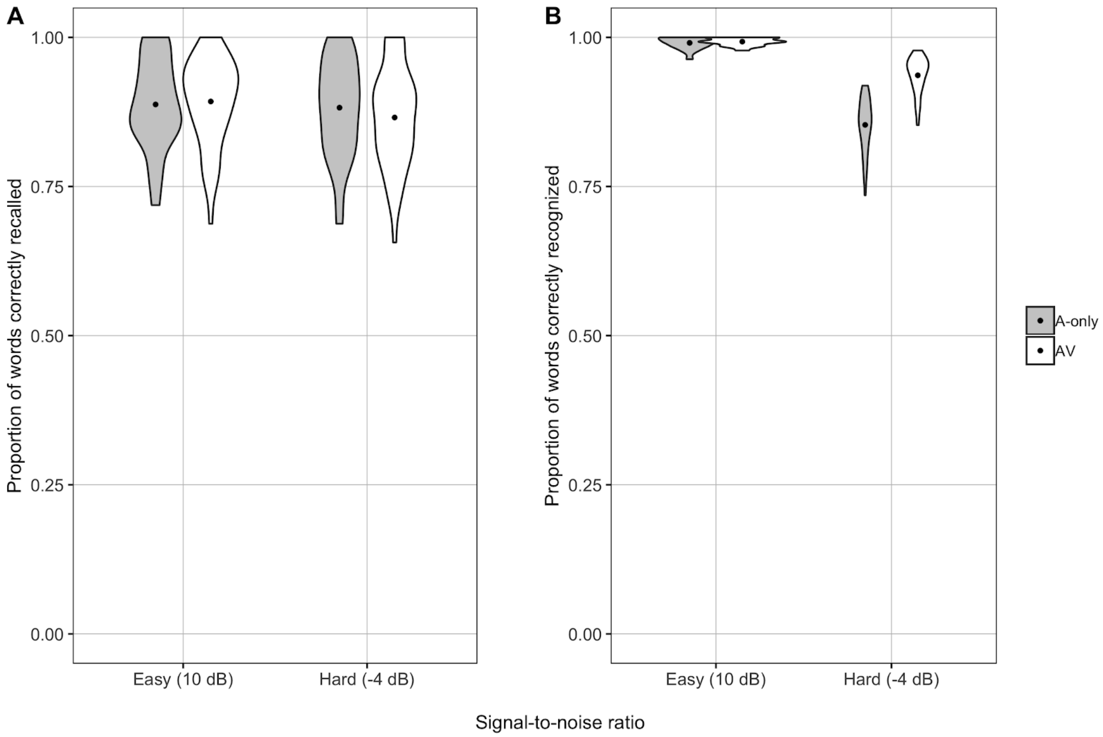

Figure 2. By-participant recall of the 2- and 3-back words (A) and overall word recognition accuracy (B) in the easy and hard SNRs in the A-only and AV modalities in Experiment S3. The dot represents the mean recall (A) or accuracy (B) in each condition, and the shape of each plot represents the distribution of responses across participants.

We compared a model with SNR, modality, lipreading ability, and the lipreading ability-by-modality interaction to a model lacking the interaction. The full model did not provide a better fit for the data (χ^2_1 = 1.71; ***p*** = 0.19); we therefore found no evidence that the effect of the visual modality on effort depends on lipreading ability. Next, we built a model including the three-way interaction between SNR, modality, and lipreading ability because it is possible that superior lipreaders have better recall in AV compared to A-only modalities, but only when the listening conditions are sufficiently difficult to necessitate the use of the visual modality. This model did not provide a better fit for the data than a model lacking the three-way interaction but including all two-way interactions (χ^2_1 = 3.47; ***p*** = 0.06). Given that these results were not significant at the pre-registered alpha-level of 0.05, we did not perform any follow up analyses to further explore this interaction.

Discussion

The conclusions of Experiment S3 are qualitatively identical to Experiment 3: participants showed poorer recall in the difficult SNR but recall performance did not differ as a function of modality, and none of the effects were moderated by lipreading.

References

Brysbaert, M., & New, B. (2009). Moving beyond Kucera and Francis: a critical evaluation of current word frequency norms and the introduction of a new and improved word frequency measure for American English. ***Behavior Research Methods***, ***41***(4), 977–990.

Picou, E. M., & Ricketts, T. A. (2014). The effect of changing the secondary task in dual-task paradigms for measuring listening effort. ***Ear and Hearing***, ***35***(6), 611–622.

Pisoni, D. B., Nusbaum, H. C., Luce, P. A., & Slowiaczek, L. M. (1985). Speech Perception, Word Recognition and the Structure of the Lexicon. ***Speech Communication***, ***4***(1-3), 75–95.
